# Ohio State Dental Society

**Published:** 1896-01

**Authors:** 


### Ohio State Dental Society.

The annual meeting of the Ohio State Dental Society was held in Columbus, on Tuesday, December 5th, and the two succeeding days; it was a very interesting and profitable meeting. There was a very good attendance; the membership not quite as large as usual, but the attendance of non-members was larger than usual, and from them there was quite an addition to the membership. A number of very good papers were read and discussed freely and fully. The subject of education was presented in rather a new aspect, in a paper by Dr. Jackman, of Cleveland, and the discussion following was of great interest.

The address of the President, Dr. Todd, presented, thoughts of special interest, which called out much discussion. Prominent among these points was that of promoting the interest of the Society—increasing its numbers, and making it more efficient. Every one present felt that he had received benefit far greater than the cost of money and time involved in coming; and five hundred more could have received like benefit had they been present, but they were not, and no one responsible but themselves.

A good part of one day was devoted to clinics, in which there were several very good things presented; among these were some electrical devices by Dr. L. E. Custer, viz: an improved electrical gold-foil annealer, which is a most admirable appliance for every one who can make the electric current available. He also presented a water rheostat for regulating the electric current; this is the most simple and efficient apparatus for this purpose we have seen, and can be afforded at a very small cost compared with the instrument commonly used for this purpose. Dr. Custer does not claim originality for this device, but he is certainly entitled to great credit for bringing it forth and presenting it in so practicable a form.

Dr. A. S. Condit, of Findlay, Ohio, illustrated a very practical method of constructing a movable bridge; the precision and efficiency of the method is better than anything heretofore presented for this purpose.

Dr. J. B. Snyder, of Bryan, Ohio, gave a clinic on the implanting of a porcelain tooth by means of a metal tube, first inserted in the gums and bone, and the post or pivot of the tooth inserted in the tube. Dr. Snyder suggested that bone material would be deposited around the tube and fix it firmly in place.

A case was exhibited, in which the tube, with a tooth in it, had been worn in the mouth for two or three months, and little or no irritation was apparent about it; during the clinic a tube was inserted at another place in the same mouth; the tube was set in a hole that had been drilled in the bone for it. While no one specially criticised the operation, it did not seem to inspire much faith in it, being so entirely contrary to former teachings and experience; if any success attends the method it will doubtless be heard from in the future.

The question of an amendment to the dental law was considered, and it was decided that an effort be made to secure such an amendment, and a committee is actively at work, and it is to be hoped will have the hearty support of the entire profession of the State.

